# Case Report: Multiple gastrointestinal stromal tumors along with numerous cutaneous neurofibromas: a case description and literature analysis

**DOI:** 10.3389/fonc.2023.1206991

**Published:** 2023-10-16

**Authors:** Congcan Zhao, Liquan Jin, Yunbo Tan, Yiming Chen, Ziting Su, Wenwu Li, Qing Yang

**Affiliations:** 1st Department of General Surgery, The First Affiliated Hospital of Dali University, Dali, Yunnan, China

**Keywords:** gastrointestinal stromal tumors, jejunum, neurofibroma, skin, neurofibromatosis type 1

## Abstract

Multiple gastrointestinal stromal tumors (GISTs) combined with cutaneous multiple neurofibromas are clinically rare. This paper presents a case of multiple gastrointestinal stromal tumors in the jejunum of a 68-year-old mother, along with her daughter who also had coexisting cutaneous multiple neurofibromas. The mother had been experiencing repeated melena for over 2 years and had previously been diagnosed with multiple small intestinal masses at other hospitals. Additionally, her 42-year-old daughter was admitted to our department due to recurrent abdominal pain caused by cholecystolithiasis. The mother and daughter both exhibited multiple nodular masses of varying sizes on their skin, including the truncus, limbs, and face, which were diagnosed as neurofibromas. The mother underwent a partial excision of the jejunum and a lateral jejunojejunal anastomosis side-to-side, as well as excision of skin lesions in our department. The final diagnosis of wild-type GISTs associated with neurofibromatosis type 1 (NF1) was confirmed through postoperative pathology, immunohistochemistry, and genetic testing results. During preoperative gastrointestinal endoscopy and intraoperative laparoscopic exploration of the gastrointestinal tract, no obvious tumors were found in her daughter. A combination of patient observations and a review of relevant literature in the field suggests that when patients present with gastrointestinal symptoms and multiple irregular painless swellings in the skin, it is important to consider the possibility of an association with NF1 and GIST. Additionally, obtaining a detailed family history can save time and improve the diagnosis of patients with both NF1 and GIST. We recommend that even if there are no gastrointestinal manifestations of GISTs in the offspring of newly mutated NF1 patients, regular review of gastroenteroscopy, imaging examination, and long-term follow-up after middle age are still crucial for the early diagnosis and treatment of NF1-related GISTs.

## Introduction

Gastrointestinal stromal tumors (GISTs) are the most common tumors derived from mesenchymal tissues in the gastrointestinal tract. It can affect the entire gastrointestinal tract but is most frequently found in the stomach (60-70%), followed by the small intestine (20-30%), colon, and esophagus. Involvement of the mesentery, greater omentum, and peritoneum is extremely rare (1–3). GISTs typically occur around the age of 60, with very few cases reported in children. There is no significant difference in occurrence between sexes (3). The majority of GISTs (approximately 85%) are accompanied by mutations in the *C-Kit* gene. The common exons for these mutations are 11 and 9, while rare mutations occur in exons 13, 17, 14, and 18. Mutations in the *PDGFRA* gene are also common, with exons 18 and 12 being frequently affected and exons 14 and 10 being less common. Autosomal dominant mutations in the *KIT* or *PDGFRA* germline are extremely rare in families (4–6). Some GISTs (10% to 15%) do not have mutations in the *C-Kit* or *PDGFRA* genes and are referred to as wild-type GISTs. However, they may be accompanied by mutations in other genes, such as the *SDH* family gene, *RAS* family gene, *BRAF*, *NF1*, and other rare genes. Depending on the presence or absence of mutation or methylation of SDH subunit genes, wild-type GISTs can be divided into two categories: *SDH*-deficient and non-*SDH*-deficient. The former category includes *SDHA* mutants, Carney triad syndrome, Carney-Stratakis syndrome, and some sporadic GISTs, which are more common in young women and predominantly occur in the stomach. The latter category mainly includes neurofibromatosis type 1 (NF1)-associated, *BRAF*-mutant, and quadruple wild-type GISTs (4–7). Among them, gastrointestinal stromal tumors (GISTs) associated with neurofibromatosis type 1 (NF1) are more commonly found in adult females. These tumors mainly occur in the small intestine and are characterized by smaller, multifocal growth. They do not exhibit *C-Kit* or *PDGFRA* mutations and have a better prognosis in terms of staging (6, 8, 9). Neurofibromatosis type 1 (NF1), also known as Reckling Hodgkin’s disease, is caused by autosomal dominant loss-of-function mutations of the *NF1* gene. The *NF1* gene is located in the 2nd band of the 11th region of the long arm of chromosome 17 and consists of 60 genetic exons spanning a length of 350 kb. It encodes neurofibrillar protein and has been reported to have more than 3000 pathogenic mutation sites (10). However, 5-10% of NF1 cases are caused by microdeletions of the entire *NF1* gene and a variable number of immediately flanking genes (11). This results in a more severe clinical phenotype. Approximately 50% of NF1 cases are inherited in families, while nearly 50% are due to *de novo* mutations. Among the inherited cases, 68.6% are maternally inherited, and 31.4% are paternally inherited (12). In addition to NF1, patients with this condition are also prone to developing other tumors, such as malignant peripheral nerve sheath tumors, optic nerve gliomas, breast cancer, leukemia, pheochromocytomas, and gastrointestinal stromal tumors (13, 14). This report presents a case of a mother with multiple jejunal gastrointestinal mesenchymal tumors and her daughter with coexisting cutaneous multiple neurofibromatosis. The patient was admitted to the Department of General Surgery I of the First Affiliated Hospital of Dali University between October 2022 and January 2023. The incidence of these gastrointestinal stromal tumors ranges from 3.9% to 25% (2, 8). However, there are limited reports on cases involving both mothers and daughters with comorbid NF1.

## Case presentation

**Figure 1:**
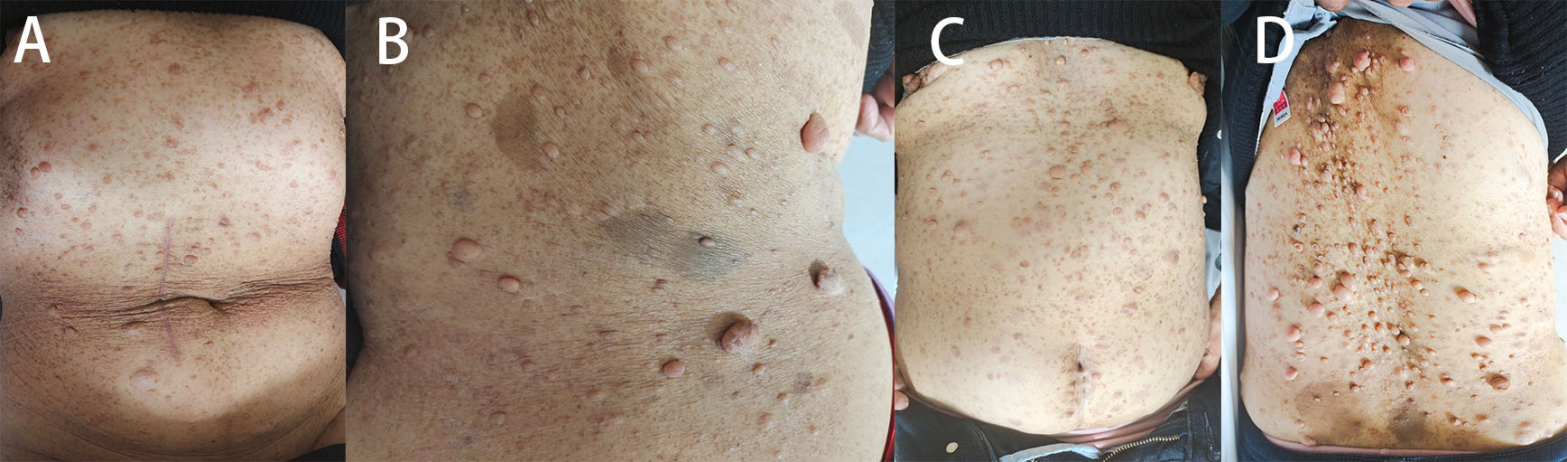
**(A, B)** Patient 1 had several skin lumps of various sizes on the back and abdomen, and there was a clear milk coffee stain that could be seen on the back. **(C, D)** Patient 2 has multiple irregular skin lumps, which are more pronounced than those of the mother.

**Figure 2:**
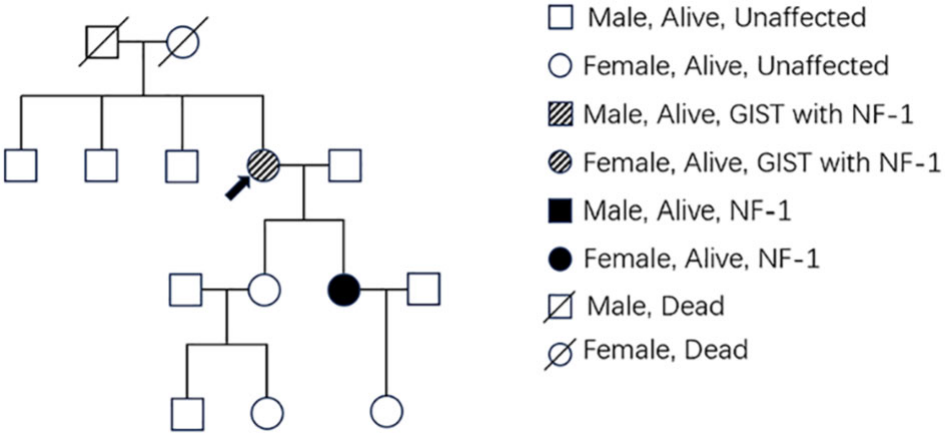
The pedigree shows family members affected with GIST or NF-1. Members with only NF1 are marked with black boxes, while those who had both NF1 and GIST are marked with deviant crease lines. The arrow indicates the patient.

**Figure 3:**
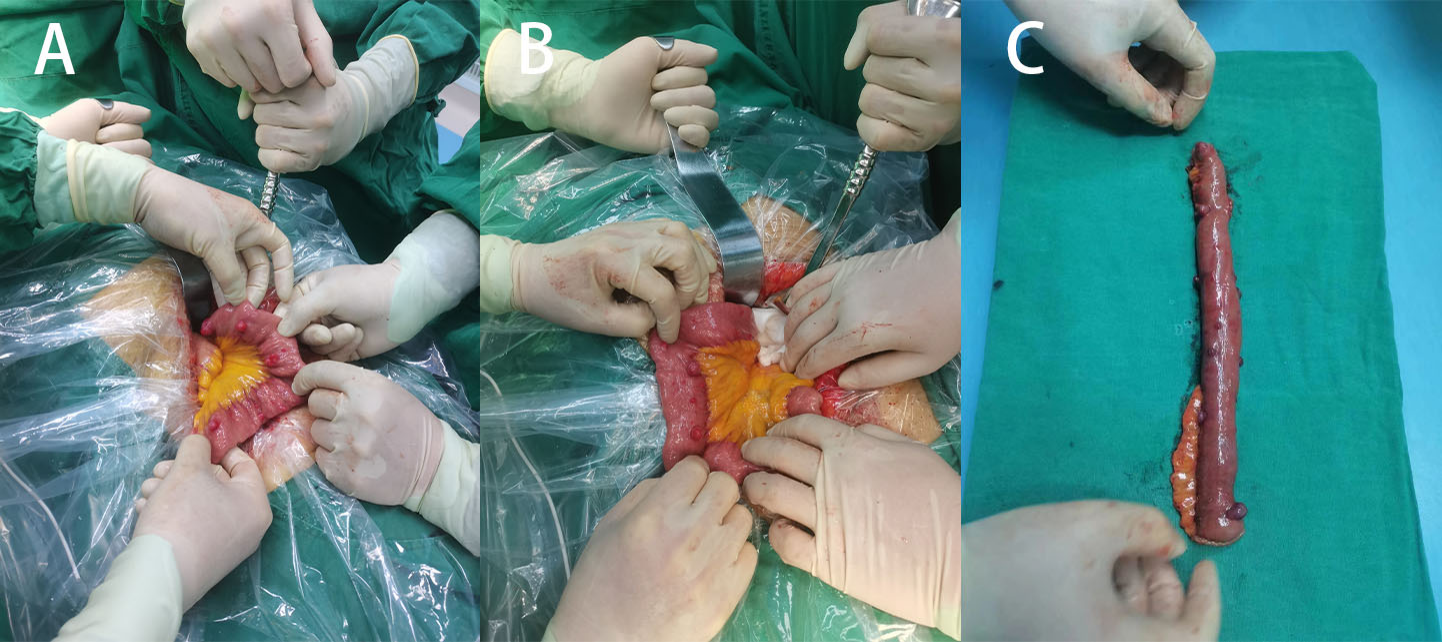
**(A)** Multiple surface tumors of the small intestine were observed during surgery (positive view). **(B)** Two large circular masses can be seen (backside view). **(C)** Surgical removal of a portion of the small intestine with a lump.

**Figure 4:**
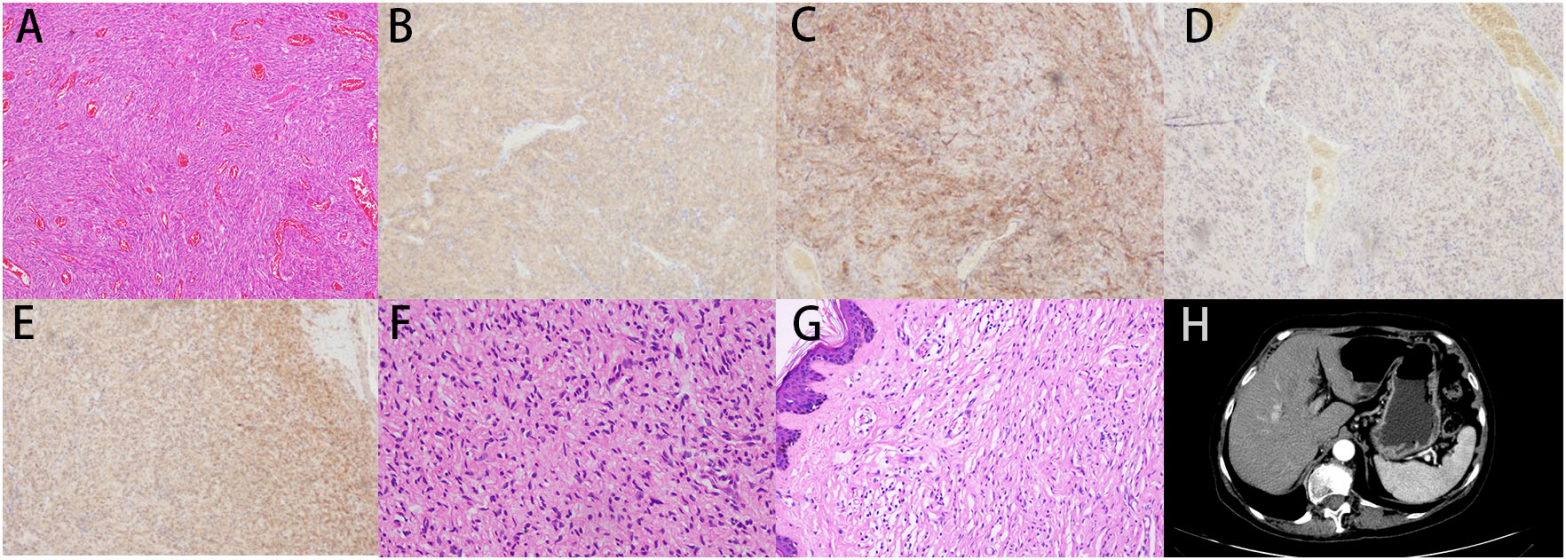
**(A)** Pathological section of intestinal mass at 100x magnification, showing numerous spindle-shaped cells. **(B)** Intestinal mass: *CD117*(+). **(C)** Intestinal mass: *CD34*(+). **(D)** Intestinal mass: *SDHB*(+). **(E)** Intestinal mass: *vimentin*(+). **(F)** Patient 1: Postoperative pathological section of skin mass, 200x. **(G)** Pathological section of the daughter’s skin tumor, 200x. **(H)** Three months later, a follow-up CT scan was performed, and the anastomosis was restored to normal. No liver metastasis was found.

Patient 1, a 68-year-old mother, has been experiencing repeated black stools for over 2 years. She also complained of fatigue and poor appetite. She visited the First People’s Hospital of Dali City, where colonoscopy and gastroscopy revealed the presence of colon polyps. A routine blood test indicated mild anemia, and she was advised to continue monitoring her symptoms. However, the black stools continued to recur during the follow-up period. As a result, the patient underwent a double balloon-enteroscopy procedure. This procedure revealed multiple protrusions in the small intestine of unknown nature and jejunal polyps (Yamada I type). Subsequently, the patient sought conservative treatment with medication at Dali Prefecture Hospital, although the specific medication used was unknown. The patient reported an improvement in the symptoms of black stool. One week ago, the patient experienced black stools again. There were no accompanying symptoms, such as abdominal distension, abdominal pain, diarrhea, nausea, vomiting, or discomfort. The patient visited our hospital for further diagnosis and treatment. Upon admission to our department, the patient was diagnosed with a’small intestinal mass’. Throughout the course of the disease, the patient’s general condition slightly deteriorated, resulting in a weight loss of approximately 5 kg. Physical examination revealed multiple nodular swellings of varying sizes on the trunk, limbs, and facial skin (**Figure 1**). These swellings were soft and nontender. There were no positive signs during cardiopulmonary or abdominal examination. The patient’s speech was fluent, mental clarity was clear, and their comprehension, calculation, and orientation were normal. Attention was focused, and the patient cooperated during the examination. The patient’s cranial nerves, muscle strength, muscle tone, tendon reflexes, and sensation were all normal, and no pathological reflexes were observed. Her parents and three brothers did not have any similar skin or gastrointestinal manifestations (**Figure 2**). She had two daughters, one of whom also had similar skin manifestations (**Figure 1**). The mother started experiencing nodular skin swellings in her 20s, while her daughter had skin manifestations since childhood. Both the mother and daughter showed a gradual increase in the size of the swellings as they aged. The mother was admitted to the hospital and underwent various tests. The test results for red blood cells (3.83×10¹²/L), hemoglobin (106 g/L), erythrocyte pressure volume (33.5 vol%), mean erythrocyte volume (87.5 fl), mean erythrocyte hemoglobin volume (27.7 pg), mean erythrocyte hemoglobin concentration (316 g/L), alpha-fetoprotein (AFP), carcinoembryonic antigen (CEA), glycoantigen 125 (CA125), glycoantigen 15-3 (CA153), glycoantigen 19-9 (CA199), and glycoantigen 72-4 (CA724) were all within the normal range. Preoperative examination and preparation were conducted prior to the surgery. Intraoperative exploration revealed multiple masses in the jejunum (**Figure 3**), leading to a partial resection of the jejunum, including the masses (**Figure 3**). A linear cutting stapler was used for side-to-side anastomosis, as per the patient’s request during the operation. Additionally, the largest cutaneous mass in the abdominal wall was excised for pathology and genetic testing. Postoperative examination revealed the following findings: 1. Multiple gastrointestinal stromal tumors were observed in the small intestine. These tumors, measuring 0.2-1.5 cm in diameter, were located in the mucosa and submucosal layer of the intestinal wall. They did not exhibit any signs of rupture and were composed of spindle-shaped cells, with rare nuclear division. Immunohistochemical staining showed positive results for CD117, CD34, SDHB, and *vimentin*. 2. A, neurofibroma was also identified in the abdominal skin mass (**Figure 4**). Mutations in *BRAF*, *ERBB2, KRAS*, *PDGFRA*, *KIT*, *NTPK1*, *NTRK2*, *NTRK3*, *FGFR1*, *FGFR2*, and *FGFR3* were not detected in the patient’s blood. Additionally, no rearrangements were found in the *NTPK1*, *NTRK2*, *NTRK3*, *FGFR1*, *FGFR2*, or *FGFR3* genes. As a result, the patient did not receive targeted drug treatment (imatinib) after surgery. Instead, she was discharged from the hospital two weeks after surgery, following symptomatic treatment including anti-infective, acid-suppressing and gastric protection, antispasmodic and analgesic, antiemetic, rehydration, and nutritional support (**Figure 2**). The patient’s recovery was satisfactory during the three-month follow-up after discharge, with no signs of tumor recurrence or metastasis observed on abdominal CT (**Figure 4**).

Patient 2, a 42-year-old woman, was admitted to our department on January 03, 2023, with a diagnosis of cholecystolithiasis with chronic cholecystitis. She had previously visited the dermatology department at other hospitals many years ago due to a’skin mass’ and underwent selective excision of some of the larger skin swellings (the patient was unsure of the specific hospital and the detailed procedure of the surgery). However, her skin swellings continued to increase in number and size, and she did not receive any further specialized treatment. Upon physical examination, the patient was in a generally acceptable condition with clear mental status, able to move independently, and cooperative during the examination. Multiple skin masses were observed all over the body (refer to **Figure 1**), with no yellowing of the skin or sclera. Cardiac examination yielded negative results, and the entire abdomen was soft with no signs of pressure pain or rebound pain. The patient exhibited normal spirit, fluent speech, and intact memory, comprehension, calculation, and orientation. There were no apparent abnormalities in the cranial nerves, muscle strength, muscle tone, or tendon reflexes. Sensory and ataxia examination yielded normal results, and pathological reflexes were absent. The spine and limbs were movable without any deformities. The routine blood results indicated a red blood cell count of 4.54×10¹²/L, a hemoglobin level of 94 g/L, an erythrocyte pressure volume of 33.5 vol%, a mean erythrocyte volume of 73.8 fl, a mean erythrocyte hemoglobin volume of 20.7 pg, and a mean erythrocyte hemoglobin concentration of 281 g/L. The blood biochemistry results showed normal levels of bilirubin, liver and kidney function, and electrolytes. Gastroscopy did not reveal any mass, while colonoscopy identified a 0.3 cm diameter polyp in the rectum. During cholecystectomy in our department, intraoperative exploration revealed obvious dilation of the common bile duct. No visible mass was found on the surface of the small intestine. Therefore, exploratory choledochotomy and cholecystectomy were performed. A 1.5x1x0.5 cm skin mass on the abdominal wall was excised and sent for pathological examination, which revealed neurofibroma (**Figure 4**). The patient declined genetic testing and was discharged from the hospital after receiving fluid infusion and other symptomatic treatments to improve the condition. She was instructed to follow up regularly after discharge.

## Methods

Patient and family consent was obtained, and relevant consent forms were signed. The patient’s blood genetic testing was conducted using OncoDrug-SeqTM by TOPGEN Bio-Testing Co Ltd. (www.hztopgen.com.cn/). The testing process involved the following steps: 1. Peripheral blood samples from patient 1 were extracted for free DNA using the QIAamp Circulating Nucleic Acid Kit(50) kit (No:55114, Qiagen, Germany), following the provided instructions. The nucleic acid concentration was measured using Qubit, and a cfDNA concentration of ≥0.1 ng/ul was considered acceptable. 2. Library construction was performed using the KAPA DNA HyperPrep Kit (No:7962312001, KAPA Biosystems, USA). First, we performed end repair and 5’ phosphorylation, as well as 3’ end A addition. Following this, a ligation reaction with a sequencing junction was carried out. Finally, PCR amplification was performed using primers containing index and UMI. The resulting product was purified to obtain prehybridized libraries with a concentration of ≥25 ng/ul and a total amount of ≥750 ng. These libraries could then be used for the next step of the experiments. Next, target genes were captured using the TargetSeqTM hybridization capture kit (lat, iGeneTech, China, www.igentech.com/). A prehybridized library with a range of 450-4000 ng was taken and incubated with a biotin-labelled specific RNA probe for hybridization. The probe and the target fragment were then captured using magnetic beads with streptavidin. Nonspecifically bound DNA fragments were washed away with a washing solution. Finally, after PCR amplification and purification, the capture library was obtained. The concentration of the capture library was required to be greater than 0.8 ng/ul. After passing the quality control, bipartite sequencing using Illumina NextSeq550 was performed to obtain the sequencing data. Using Dingjing’s self-constructed analysis process, the HG19 version was used as the reference genome for Sangshin’s analysis process. The analysis involved a probe corresponding to a region of approximately 78 Kbp size as the bed file, and it included quality control (**Table 1**), cleaning, comparison, and analysis of three aspects of the target genes: SNV, InDel mutation, and gene rearrangement (**Tables 2**, **3**).

**Table 1:** Illumina NextSeq550 sequencing quality control information.

Quality control project	Quality control scope	Quality control data	Quality control results
Sequencing quality value Q30	≥ 80%	91.73%	Qualified
Depth of sequencing	Organizational samples>500x ctDNA samples >1000x	21834x	Qualified
Coverage of sequencing	≥ 95%	99.51%	Qualified
Purity of tumor cell content	A-B-C	/	/

Tumor cell content rating, A: ≥ 20%; B: 5%-20%; C: <5%. Samples such as blood, cfDNA, wax rolls, unclear cancer types, extensive tissue necrosis, and insufficient sample size are unable to evaluate tumor cell content.

**Table 2:** Gene testing shows single base mutations and small insertion deletions in genes.

Gene	Variant type	cDNA change	Amino acid changes	Exon	transcript	Bundance of variation
*BRAF*	Single base mutation and deletion of small fragments were not detected	/	/	/	/	/
*ERBB2*	Single base mutation and deletion of small fragments were not detected	/	/	/	/	/
*KRAS*	Single base mutation and deletion of small fragments were not detected	/	/	/	/	/
*PDGFRA*	Single base mutation and deletion of small fragments were not detected	/	/	/	/	/
*KIT*	Single base mutation and deletion of small fragments were not detected	/	/	/	/	/
*NTRK1*	Single base mutation and deletion of small fragments were not detected	/	/	/	/	/
*NTRK2*	Single base mutation and deletion of small fragments were not detected	/	/	/	/	/
*NTRK3*	Single base mutation and deletion of small fragments were not detected	/	/	/	/	/
*FGFR1*	Single base mutation and deletion of small fragments were not detected	/	/	/	/	/
*FGFR2*	Single base mutation and deletion of small fragments were not detected	/	/	/	/	/
*FGFR3*	Single base mutation and deletion of small fragments were not detected	/	/	/	/	/

**Table 3:** Gene testing and gene rearrangement results.

Gene	Fusion partner	Fusion gene transcript	Exon	Abundance of variation
*NTRK1*	No gene rearrangement was detected	/	/	/
*NTRK2*	No gene rearrangement was detected	/	/	/
*NTRK3*	No gene rearrangement was detected	/	/	/
*FGFR1*	No gene rearrangement was detected	/	/	/
*FGFR2*	No gene rearrangement was detected	/	/	/
*FGFR3*	No gene rearrangement was detected	/	/	/

## Discussion

The incidence of gastrointestinal stromal tumors is approximately 1 to 1.5 per 100,000 people per year (15). In the early stage, there are no clinical manifestations. However, in the middle and late stages, symptoms such as early satiety, dysphagia, abdominal distension, obstruction, epigastric pain, vomiting of blood or black stools, and self-palpation of an abdominal mass may occur, along with other digestive symptoms. These symptoms can be accompanied by anemia, weight loss, liver metastasis, abdominal implantation spread metastasis, etc. In some cases, gastrointestinal stromal tumors are incidentally discovered during physical examinations or while diagnosing and treating other diseases (16). Endoscopy and ultrasound endoscopy (EUS) play a crucial role in diagnosing GISTs, especially for GISTs with a diameter of less than 2 cm. These techniques can detect malignant features such as irregular tumor margins, ulcer formation, and cystic changes. Additionally, EUS can guide puncture biopsy procedures. CT, particularly enhanced CT, is the preferred diagnostic tool for GIST, as well as for evaluating and monitoring the condition. Benign foci typically appear as lesions with a diameter of less than 5 cm, clear boundaries, homogeneous density, and calcification. On the other hand, malignant lesions exhibit contrasting characteristics. MRI offers excellent tissue resolution and is advantageous for GISTs in specific locations without exposing the patient to ionizing radiation. Benign lesions generally display regular morphology and relatively uniform signals, while most malignant lesions show the opposite. PET-CT scanning is recommended for the early evaluation of targeted drug efficacy, tumor resistance, and recurrence (17). In this case, the mother presented with gastrointestinal bleeding, anemia, and weight loss as her main symptoms. However, she did not exhibit any other symptoms, making the preliminary diagnosis challenging. Gastroscopy was performed to rule out the stomach as the most common site of GIST. The patient’s resection specimen revealed that the tumor was located in the beginning of the jejunum and was less than 2 cm in size. This made it difficult to detect the tumor using enhanced CT and ultrasound. To further confirm the diagnosis, the patient underwent a double balloon-enteroscopy examination. This examination revealed multiple bulges at the beginning of the jejunum, leading to the initial consideration of a gastrointestinal stromal tumor. GISTs can be classified into different types based on their growth mode, including submucosal, intramural, subserosal, and extragastrointestinal types. Typically, GISTs are solitary tumors with well-defined boundaries within the intestinal wall. However, in this particular case, both intraoperative and postoperative pathology revealed the presence of multiple tumors in the small intestine, primarily located in the mucosal and submucosal layers of the intestinal wall. This observation suggests that the tumors were growing at an accelerated rate, which could have resulted in necrosis and shedding. Consequently, the patient experienced recurrent gastrointestinal bleeding, black stools, and anemia. These symptoms can be attributed to the rapid growth and subsequent complications of the tumors. Gene mutation analysis is crucial in planning treatment with targeted agents such as tyrosine kinase inhibitors. The latest 2020 World Health Organization (WHO) STS and Osteosarcoma Classification codes classify all GISTs as malignant for the purpose of classification (18). GISTs are less likely to metastasize, with the liver and peritoneum being common sites of metastasis and lymph node metastasis being rare (9, 19). Approximately 30% of GISTs are malignant, and the 5-year postoperative survival rates range from 50% to 65%, with recurrence or metastasis occurring in 40% to 90% of operated patients (19, 20). Prognostic factors such as tumor site, size, nuclear schizophrenia, and the presence of rupture are important considerations. The treatment of GIST typically involves surgery and systemic therapy. Surgery is usually the initial choice for localized tumors. For smaller GISTs (less than 2 cm in diameter), the surgical approach can vary depending on the tumor’s location, with options including laparoscopic surgery, endoscopic resection, or open surgery. In cases where the tumor is larger and difficult to remove due to the involvement of nearby organs, neoadjuvant therapy may be considered if the tumor is responsive to TKIs (21). During surgery, it is important to avoid tumor rupture, and lymph node dissection is generally not recommended. Our case report focuses on a patient who underwent surgery as the most effective treatment due to gastrointestinal bleeding and a tumor diameter of less than 2 cm. Postoperative treatment for GIST often involves adjuvant therapy, with imatinib being the first-line therapeutic agent for locally advanced, inoperable, and metastatic patients, as well as for surgical adjuvant therapy. This drug is effective in treating most GISTs (22, 23), except for GISTs without *KIT/PDGFRA* mutations. Additionally, avatinib, sunitinib, and rosutinib are available as second-, third-, and fourth-line drugs. The patient underwent one genetic test, which did not detect any mutations in *KIT* or *PDGFRA*. Since the mother did not receive tyrosine kinase inhibitor treatment, regular follow-up was crucia. Three months after surgery, the reexamination showed no signs of recurrence, but long-term re-examination and observation are necessary. Some patients may require chemotherapy-assisted therapy.

NF1 is the most common form of neurofibromatosis, accounting for approximately 90% of cases (24). It has a global prevalence of approximately 1 case per 3,000 people on average. NF1 patients commonly experience symptoms in childhood, including multiple neurofibromas (such as skin-type neurofibromas and plexiform neurofibromas), coffee milk spots, freckles, optic nerve gliomas, and damage to affected tissues and organs (such as scoliosis and long bone dysplasia). Cognitive impairment, attention deficit hyperactivity disorder, and autism spectrum disorders are also observed. Neurofibromas, particularly skin-type neurofibromas, are a typical manifestation of this disease (25). Additionally, patients with NF1 are at a higher risk of developing neurological tumors such as optic nerve gliomas, glioblastomas, malignant peripheral nerve sheath tumors, and rhabdomyosarcomas. The presentation of symptoms can vary significantly among NF1 patients. The diagnosis of patients with NF1 is currently based on the recommendations of the 2021 International Neurofibromatosis Diagnostic Criteria Consensus Group, which revised the 1987 diagnostic criteria for NF1 (26). According to these criteria, individuals with a family history of NF1 must fulfil one or more clinical features, while those without a family history of NF1 must fulfil two or more clinical features, Additionally, patients need to be evaluated for associations with Legius syndrome, McCune–Albright syndrome, II syndrome, other neurological conditions, and the presence of a neurological tumor. Genetic testing is highly valuable for diagnosis, but unfortunately, there is currently no cure for NF1. Symptomatic treatment is the main approach, with invasive treatments being commonly used. For instance, corrective surgery is often needed to fuse abnormal vertebrae in cases of combined dystrophic scoliosis. Other surgical procedures include the removal of cutaneous neurofibromas through excision, laser ablation, and electrodesiccation for smaller lesions. Pain management and surgical excision are options for treating plexiform neurofibromas. Brace fixation is recommended for patients with long bone dysplasia, while children with neurogliomas causing neurological symptoms or signs may undergo shunt placement, surgical excision, or chemotherapy. Regarding drug treatments, many targeted therapeutic agents that act on the *RAS* signaling pathway are currently being evaluated in preclinical trials. NF1 has a clear familial history. In our reported case, we observed that if the mother had NF1, there was a 50% likelihood of the next generation also having NF1. While both the mother and daughter had NF1, only the mother had GISTs. The daughter, on the other hand, exhibited more obvious skin symptoms but had a higher risk of other tumors. Therefore, it is recommended to assess the frequency of NF1 in patients with NF1 compared to those without NF1. Additionally, patients with NF1 should be encouraged to undergo genetic testing to guide disease management.

**Figure 5:**
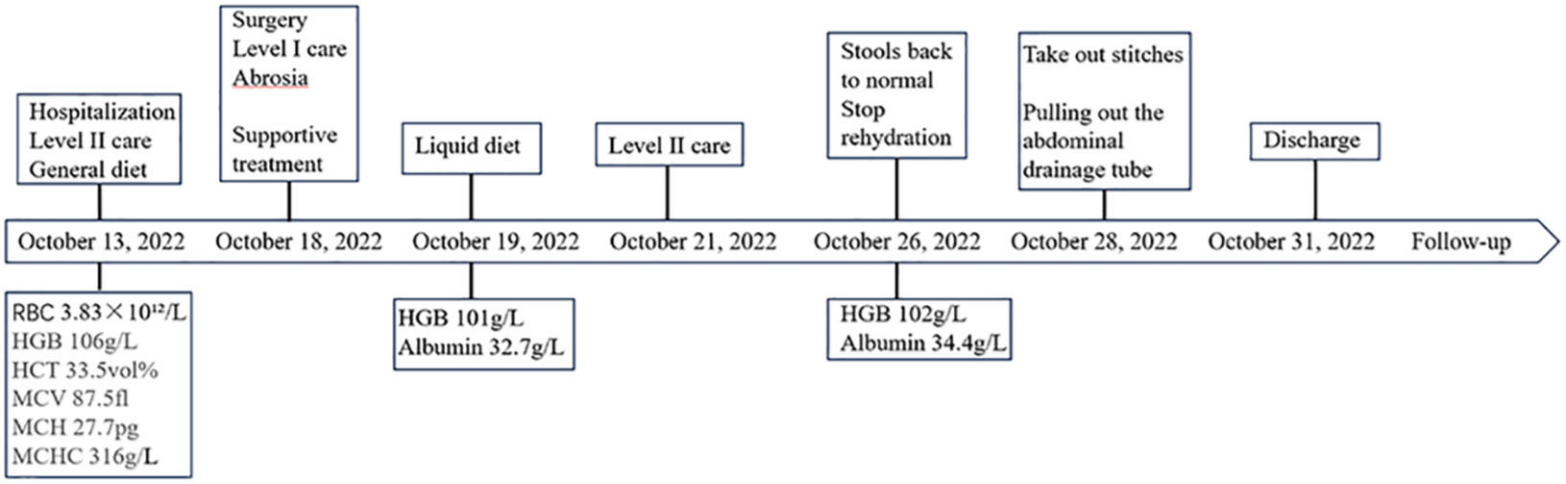
The timeline for treatment regimens.

When reviewing the course of treatment in this case (**Figure 5**), it was observed that individuals with NF1 have a 2- to 5-fold increased risk of malignant tumors compared to the general population. Additionally, these individuals have a 50-fold increased risk of high-grade tumors (27). Therefore, early detection of potentially treatable complications is crucial. While there have been literature reports of GISTs associated with NF1 (**Table 4**), the early diagnosis in mothers remains unclear. This serves as a reminder for clinicians to pay attention to the family history when assessing the relationship between NF1 and GISTs in patients presenting with typical manifestations of NF1 skin and gastrointestinal symptoms. It is crucial to consider the presence of gastrointestinal stromal tumors in middle-aged patients with a family history of NF1 when gastrointestinal symptoms arise. Early and prompt diagnosis and treatment, along with regular gastroenteroscopy or imaging reviews, are essential.

**Table 4:** GIST instances connected to NF1 that have been reported between 2000 and 2022.

Author	Report type	Title
Schaeffer, H. D.,etc.	Case report	Neurofibromatosis-associated Gastrointestinal Stromal Tumor Causing Small Bowel Obstruction
Bharath, B. G., etc.	Case report	Challenges in the management of metastatic gastrointestinal stromal tumor in a patient with neurofibromatosis type 1: a case report
Xu, H. Y., etc.	Case report	Multiple Gastrointestinal Stromal Tumor with Neurofibromatosis Type I:Report of One Case
Tran, T. A. N., etc.	Case report	Concomitant neuroendocrine tumor and gastrointestinal stromal tumor in a duodenal fine needle aspiration: A cytologic clue for neurofibromatosis type 1
Sooklal, S., etc.	Case report	Neurofibromatosis Type 1-Associated Gastrointestinal Stromal Tumor
Mandalà, S., etc.	Case report	Small bowel gastrointestinal stromal tumor presenting with gastrointestinal bleeding in patient with type 1 Neurofibromatosis: Management and laparoscopic treatment. Case report and review of the literature
Koffi, G. M., etc.	Case report	Gastrointestinal Stromal Tumor Associated with Neurofibromatosis Type 1 Simulating a Neurofibrosarcoma in a Black African Adult Patient
Arikan, S., etc.	Case report	Giant composite pheochromocytoma and gastrointestinal stromal tumor in a patient with neurofibromatosis: A case report
Arif, A. A., etc.	Case report	Pancreatic Gastrinoma, Gastrointestinal Stromal Tumor (GIST), Pheochromocytoma, and Hürthle Cell Neoplasm in a Patient with Neurofibromatosis Type 1: A Case Report and Literature Review
Minkiewicz, I., etc.	Case report	CO-OCCURRENCE OF ADRENOCORTICAL CARCINOMA AND GASTROINTESTINAL STROMAL TUMOR IN A PATIENT WITH NEUROFIBROMATOSIS TYPE 1 AND A HISTORY OF ENDOMETRIAL CANCER
Bono, D., etc.	Case report	Association of right breast cancer and ileal gastrointestinal stromal tumor in a patient with type I neurofibromatosis: Case report and review of the literature
Park, E. K., etc.	Case report	Synchronous Gastrointestinal Stromal Tumor and Ampullary Neuroendocrine Tumor in Association with Neurofibromatosis Type 1: A Report of Three Cases
Kou, Y. W., etc.	Case report	KIT and platelet-derived growth factor receptor α wild-type gastrointestinal stromal tumor associated with neurofibromatosis type 1: Two case reports
Kalayanamitra, R., etc.	Case report	The Bleeding Bowel: A Rare Case of Neurofibromatosis Type 1-associated Gastrointestinal Stromal Tumor in a Young Male
Graur, F., etc.	Case report	EPCephalic duodenopancreatectomy for neurofibromatosis associated with gastrointestinal stromal tumor. A case report
Shamila, M. A., etc.	Case report	Duodenal neuroendocrine tumor, adenocarcinoma and gastrointestinal stromal tumor in association with neurofibromatosis type 1: An unique occurrence
Lee, J. M., etc.	Case report	Intraductal papillary bile duct adenocarcinoma and gastrointestinal stromal tumor in a case of neurofibromatosis type 1
Hammami, A., etc.	Case report	Gastrointestinal stromal tumor (GIST) inpatient with Von Recklinghausen’s disease
Al Momani, L. A., etc.	Case report	Recurrent Gastric Gastrointestinal Stromal Tumor in a Patient with Neurofibromatosis
Abdessayed, N., etc.	Case report	Rare triad of periampullary carcinoid, duodenal gastrointestinal stromal tumor and plexiform neurofibroma at hepatic hilum in neurofibromatosis type 1: a case report
Yamamoto, R., etc.	Case report	The Coexistence of Somatostatinoma and Gastrointestinal Stromal Tumor in the Duodenum of a Patient with Von Recklinghausen’s Disease
Yamada, Y., etc.	Case report	Neuroendocrine Tumor of the Ampulla of Vater and Gastrointestinal Stromal Tumor of the Duodenum in a Patient with Von Recklinghausen’s Disease
James, A. W., etc.	Case report	Coincident liposarcoma, carcinoid and gastrointestinal stromal tumor complicating type 1 neurofibromatosis: Case report and literature review
Chen, Y. Y., etc.	Case report	Gastrointestinal stromal tumor mimicking ovarian malignancy in a woman with type I neurofibromatosis
Kim, S. B., etc.	Case report	Massive upper gastrointestinal bleeding from multiple gastrointestinal stromal tumor in a neurofibromatosis patient
Swain, S. K., etc.	Case report	Unusual presentation of gastrointestinal stromal tumor of stomach in neurofibromatosis type 1: a case report
Sawalhi, S., etc.	Case report	Behavior of advanced gastrointestinal stromal tumor in a patient with von-Recklinghausen disease: Case report
Malhotra, A., etc.	Case report	Extra Gastrointestinal Stromal Tumor treated with imatinib in a patient with Neurofibromatosis type 1
Aslan, F., etc.	Case report	Gastrointestinal stromal tumor perforation in a case with neurofibromatosis presenting with abdominal pain
Takeuchi, H., etc.	Case report	Synchronous double tumor of breast cancer and gastrointestinal stromal tumor in a patient with neurofibromatosis type 1: report of a case
Namgung, H., etc.	Case report	Gastrointestinal stromal tumor with KIT mutation in neurofibromatosis type 1
Day, F. L., etc.	Case report	Neurofibromatosis type 1-associated wild-type gastrointestinal stromal tumor treated with anti-IGF-1R monoclonal antibody
Ohtake, S., etc.	Case report	Duodenal gastrointestinal stromal tumor resembling a pancreatic neuroendocrine tumor in a patient with neurofibromatosis type I (von Recklinghausen’s disease): a case report
Millonig, G., etc.	Case report	Gastrointestinal stromal tumor in a patient with undiagnosed neurofibromatosis type I: an uncommon cause of extrahepatic cholestasis
Kitagawa, M., etc.	Case report	Gastrointestinal Stromal Tumor in a Patient with Neurofibromatosis: Abscess Formation in the Tumor Leading to Bacteremia and Seizure
Takeuchi, H., etc.	Case report	Duodenal Gastrointestinal Stromal Tumor Treated by Wedge Resection in a Patient with Neurofibromatosis Type 1: Report of a Case and Review of the Japanese Literature
García, A. B., etc.	Case report	Gastrointestinal bleeding secondary to gastrointestinal stromal tumor in two patients with neurofibromatosis
Chetty, R., etc.	Case report	Vasculopathic changes, a somatostatin-producing neuroendocrine carcinoma and a jejunal gastrointestinal stromal tumor in a patient with type 1 neurofibromatosis
Beltrán, M. A., etc.	Case report	Gastrointestinal stromal tumor (GIST) in a patient with neurofibromatosis type 1. Report of one case
Aboutaleb, E., etc.	Case report	c-KIT positive Gastrointestinal Stromal Tumor presenting with acute bleeding in a patient with neurofibromatosis type 1: a case report
Liu, T., etc.	Case report	A gastrointestinal stromal tumor of the stomach morphologically resembling a neurofibroma: demonstration of a novel platelet-derived growth factor receptor alpha exon 18 mutation
Koshariya, M., etc.	Case report	Gastrointestinal stromal tumor of gastrohepatic omentum in a patient with von Recklinghausen’s disease (neurofibromatosis type 1
Kalender, M., etc.	Case report	Effect of sunitinib on metastatic gastrointestinal stromal tumor in patients with neurofibromatosis type 1: a case report
Han, S. H., etc.	Case report	Malignant gastrointestinal stromal tumor in a patient with neurofibromatosis type 1
Bettini, R., etc.	Case report	Ampullary somatostatinomas and jejunal gastrointestinal stromal tumor in a patient with Von Recklinghausen’s disease
Nemoto, H., etc.	Case report	Novel NF1 gene mutation in a Japanese patient with neurofibromatosis type 1 and a gastrointestinal stromal tumor
Juergens, K. U., etc.	Case report	Duodenal somatostatinoma and gastrointestinal stromal tumor associated with neurofibromatosis type 1: diagnosis with PET/CT
Wang, S. M., etc.	Case report	Spontaneous rupture of recurrent gastrointestinal stromal tumor associated with neurofibromatosis type 1
Kramer, K., etc.	Case report	GI hemorrhage with fulminant shock induced by jejunal gastrointestinal stromal tumor (GIST) coincident with duodenal neuroendocrine carcinoma (NET) + neurofibromatosis (NF) – case report and review of the literature
Sinha, R., etc.	Case report	Mesenteric gastrointestinal stromal tumor in a patient with neurofibromatosis
Nakamura, Y., etc.	Case report	A case of von Recklinghausen disease accompanied with duodenal gastrointestinal stromal tumor and carcinoma in adenoma of the sigmoid colon
Nakamura, H., etc.	Case report	A case of upper jejunal gastrointestinal stromal tumor (GIST) accompanied with von Recklinghausen’s disease

## Conclusions

A combination of this case and a review of the relevant literature in this field suggests that in patients presenting with gastrointestinal symptoms and multiple irregular painless skin masses, it is important to consider the association between NF1 and GISTs, although a definitive diagnosis may be challenging. It is recommended to obtain a detailed family history, as this information could potentially save time and improve outcomes in diagnosing patients with both NF1 and GISTs. Additionally, we propose that regular review of gastroenteroscopy, imaging examination, and long-term follow-up after middle age should be conducted for the offspring of newly mutated NF1 patients, even in the absence of gastrointestinal manifestations of GISTs. This approach is crucial for the early diagnosis and treatment of NF1-related GISTs.

## Data availability statement

The datasets presented in this study can be found in online repositories. The names of the repository/repositories and accession number(s) can be found in the article/supplementary material.

## Ethics statement

The studies involving humans were approved by The independent Ethics Committee of the First Affiliated Hospital Center of Dali University. The studies were conducted in accordance with the local legislation and institutional requirements. The participants provided their written informed consent to participate in this study. Written informed consent was obtained from the individuals for the publication of any potentially identifiable images or data included in this article.

## Author contributions

LJ initiated the research and drafted the manuscript. CZ drafted the manuscript. YT, YC and ZS took care of the patient and performed the surgery. WL analyzed the data and assisted in the preparation of the manuscript. QY checked the language issues. All authors contributed to the article and approved the submitted version.
